# Tobacco quitline performance: Comparing the impacts of early cessation and proactive re-engagement on callers’ smoking status at follow-up at 12 months

**DOI:** 10.18332/tid/159125

**Published:** 2023-02-15

**Authors:** Daniel G. Cassidy, Xin-Qun Wang, Indika Mallawaarachchi, Kara P. Wiseman, Jon O. Ebbert, John A. Blue Star, Chase A. Aycock, Rosemary Estevez Burns, John R. Jones, Andrea E. Krunnfusz, Jennifer P. Halbert, Natalie M. Roy, Jordan M. Ellis, Juinell B. Williams, Robert C. Klesges, Gerald W. Talcott

**Affiliations:** 1 Wilford Hall Ambulatory Surgical Center, Joint Base San Antonio, Lackland AFB, Texas, United States; 2 Department of Public Health Sciences, School of Medicine, University of Virginia, Charlottesville, United States; 3 Mayo Clinic Comprehensive Cancer Center, Mayo Clinic, Rochester, United States; 4 Department of Psychology, East Carolina University, Greenville, United States

**Keywords:** abstinence, quitline, nicotine replacement therapy, tobacco cessation intervention, proactive re-engagement

## Abstract

**INTRODUCTION:**

While tobacco Quitlines are effective in the promotion of smoking cessation, the majority of callers who wish to quit still fail to do so. The aim of this study was to determine if 12-month tobacco Quitline smoking cessation rates could be improved with re-engagement of callers whose first Quitline treatment failed to establish abstinence.

**METHODS:**

In an adaptive trial, 614 adult smokers, who were active duty, retired, and family of military personnel with TRICARE insurance who called a tobacco Quitline, received a previously evaluated and efficacious four-session tobacco cessation intervention with nicotine replacement therapy (NRT). At the scheduled follow-up at 3 months, callers who had not yet achieved abstinence were offered the opportunity to re-engage. This resulted in three caller groups: 1) those who were abstinent, 2) those who were still smoking but willing to re-engage with an additional Quitline treatment; and 3) individuals who were still smoking but declined re-engagement. A propensity score-adjusted logistic regression model was generated to compare past-7-day point prevalence abstinence at 12 months post Quitline consultation.

**RESULTS:**

Using a propensity score adjusted logistic regression model, comparison of the three groups resulted in higher odds of past-7-day point prevalence abstinence at follow-up at 12 months for those who were abstinent at 3 months compared to those who re-engaged (OR=9.6; 95% CI: 5.2–17.8; Bonferroni adjusted p<0.0001), and relative to those who declined re-engagement (OR=13.4; 95% CI: 6.8–26.3; Bonferroni adjusted p<0.0001). There was no statistically significant difference in smoking abstinence between smokers at 3 months who re-engaged and those who declined re-engagement (OR=1.39; 95% CI: 0.68–2.85).

**CONCLUSIONS:**

Tobacco Quitlines seeking to select a single initiative by which to maximize abstinence at follow-up at 12 months may benefit from diverting additional resources from the re-engagement of callers whose initial quit attempt failed, toward changes which increase callers’ probability of success within the first 3 months of treatment.

**TRIAL REGISTRATION:**

This study is registered at clinicaltrials.gov (NCT02201810).

## INTRODUCTION

Tobacco is the single most preventable cause of death in the US^1^, and veterans of the US Military use tobacco products at a rate higher than the general US population^2^. In 2016, the Secretary of Defense issued a memorandum^3^ calling for action to address the high prevalence of tobacco use in the military. The memo mandated dramatic increases in the cost of tobacco products in military stores, establishing price parity with historically more expensive off-base establishments, and called for improved and expanded tobacco cessation programs.

The Military Health System serves over 9.6 million retirees, family members, and active-duty military members living and working in countries around the world^4^. Consequently, the Defense Health Agency-administered health system ‘TRICARE’ shares with national and state public health interests a common challenge germane to tobacco control: how best to reach millions of smokers seeking to quit. Available virtually anywhere where there is a phone service, tobacco Quitlines offer a compelling combination of reach and effectiveness^5^. Proactive Quitlines (i.e. those which pre-schedule sessions and then call participants on the appointed day and time) produce better outcomes relative to reactive Quitlines (i.e. those in which participants must call to initiate each interaction)^6^; and this general effect has been replicated with active-duty military and TRICARE beneficiaries, for whom proactive treatment has doubled the odds of abstinence from tobacco at the follow-up at 12 months^7^. But even with the aid of proactive treatment, the majority of those who desired to quit smoking failed to do so, and half of the smokers who quit during treatment had re-initiated by the follow-up at 12 months^7^. The decay of tobacco cessation treatment effects over time is firmly established in the tobacco control literature^8,9^. As a result, a growing body of research has developed on how to best re-engage Quitline callers whose initial quit attempt was not successful.

Emerging evidence suggests that re-engagement efforts made by Quitlines function to increase the probability that participants will make subsequent quit attempts. Vickerman et al.^10^ , for example, observed a five-fold increased odds of re-engagement as a consequence of proactive telephone outreach by Quitline staff between one and three months after the initial quit attempt. More extensive literature, dating back nearly 10 years, characterizes phone contact as effective in re-engaging 12–28% of Quitline participants who failed a previous quit attempt^10-12^. Still unclear, by contrast, is the degree to which re-engagement assists callers following an unsuccessful quit attempt. Characterization of the effectiveness of such re-engagement efforts will assist Quitlines in determining how best to allocate finite personnel and financial resources.

The current study sought to address this gap in the literature by determining the degree to which Quitline abstinence rates can be improved with re-engagement of callers whose first Quitline treatment failed to establish abstinence. To this end, we compared tobacco use rates at 12 months across three groups of participating TRICARE beneficiaries who called the ‘Freedom Quitline’: 1) those who were abstinent at check-in at 3 months; 2) those who were still smoking at 3 months but accepted an invitation to re-engage with proactive tobacco Quitline treatment; and 3) individuals who were still smoking at 3 months but declined the opportunity to re-engage for a second Quitline treatment. We were interested in how re-engagement at 3 months improved outcomes at follow-up at 12 months. We were also interested in how participants who re-engaged compared at 12 months with those who had either reported abstinence at the check-up at 3 months or were still smoking but declined the opportunity to re-engage.

## METHODS

### Design

This adaptive trial is a secondary analysis of data which derive from a randomized controlled trial designed to determine the effect of three re-engagement strategies on long-term smoking cessation^13^. Participants were those who were still smoking at 3 months following an initial Quitline intervention composed of counseling and nicotine replacement therapy. Smokers who were willing to try again were randomized into three re-engagement conditions: rate reduction, repetition of the initial treatment, or their choice of the preceding treatments. Quitline interventionists were research specialists trained to provide tobacco cessation treatment as part of the National Heart, Lung, and Blood Institute (NHLBI; HL123978). Dubbed the ‘Freedom Quitline’, this service provided smoking cessation treatment exclusively to TRICARE beneficiaries from 2017 through 2020 (Supplementary file).

### Participants

Recruited participants were 614 adult TRICARE beneficiaries, including active-duty and retired military personnel from the Army, Navy, Air Force, Marines, and Coast Guard, and those among their family members covered under their insurance. Participants were recruited with physical and electronic media including posters, flyers and business cards at medical and dental facilities on military bases and through websites including TRICARE.mil, Military. com, thewingmantoolkit.org, Health Net Federal Services (hnfs.org), soldierforlife.com, 79 mdw.af.mil, and UCANQUIT2.org. Eligible participants were aged ≥18 years, had smoked at least five cigarettes daily over the preceding year, and were willing to make a quit attempt in the next 30 days. Because participation entailed access to nicotine replacement therapy (NRT), individuals who were pregnant, breastfeeding, or planning to become pregnant in the next 12 months, those with a pre-identified allergy to nicotine, or with an unstable heart condition, were excluded from the study. All persons participating in this study gave informed consent prior to engaging with the protocol.

### Measures

All measures were administered by telephone at baseline, at 3 months, and at 12 months from the enrollment date. Demographic variables included age, gender, race, ethnicity, education level, military status, and marital status. Smoking-related variables included intensity of physical addiction to nicotine, evaluated using the Fagerström test for nicotine dependence (FTND)^14^. Other smoking-related variables included number of cigarettes smoked per day, number of years smoked, lifetime number of quit attempts, and use of NRT and additional prescription smoking cessation aids, each of which was queried directly. Past-7-day point prevalence, the primary outcome measure, was defined as the self-reported absence of tobacco use within the seven days preceding follow-up at 3 months and at 12 months^15^, as determined over the phone by study the research specialists.

### Intervention

All participants engaged in an initial series of four proactively initiated telephone smoking cessation calls executed in conjunction with mailing of an 8-week supply of 7, 14 or 21 mg nicotine patch therapy (contingent upon baseline smoking rate). The initial four calls were structured to include in sequence: 1) rapport-building, the evocation of change talk, and rate reduction at the first contact; 2) NRT use, the management of smoking-related triggers, and discussion of quit date; 3) post-quit problem solving, management of withdrawal symptoms, and short-term relapse prevention; and 4) long-term relapse prevention planning. Contingent upon randomization, re-engagement of those not abstinent at the follow-up at 3 months comprised either repetition of the initial call content or an alternative sequence that omitted establishment of a quit date, but which included: 1) the evocation of change talk, use of NRT, a review of rate reduction strategies, and encouragement to reduce daily cigarettes by at least 25%; 2) NRT use, the management of smoking-related triggers, and a recommended further 25% reduction in daily cigarettes; and 3) review of strategies for sustained rate reduction and preparation for a quit attempt when ready.

Counselors participating in the study were research specialists hired by the University of Tennessee Health Science Center who received extensive training both in the intervention components elaborated above and in motivational interviewing, including a two-day workshop and weekly skills-training. Calls were audio-recorded and subject to random fidelity checks which formed the basis for each counselor’s monthly individual feedback concerning intervention delivery and their interactions with Quitline callers. Initial motivational interviewing proficiency and subsequent fidelity checks were accomplished with reference to the Motivational Interviewing Treatment Integrity Code (MITI 3.1.1)^16^. This protocol was approved by the Institutional Review Board (IRB) of the 59th Medical Wing, Joint Base San Antonio-Lackland, Texas.

### Power calculations

Detailed power calculations for the primary analysis of the parent study were described in Little et al.^13^. The current study was a secondary analysis comparing smoking cessation rates at 12 months among those who quit or were still smoking at 3 months (some of whom elected to re-engage with Quitline treatment), thus sample size calculations for this secondary analysis are not required.

### Statistical analyses

To reduce potential treatment selection bias (given that each participant was still smoking at 3 months, was offered re-engagement but free to decline) and control for the pretreatment (i.e. before the second-phase treatment) imbalances on participants’ characteristics, propensity score methods were developed to minimize or eliminate selection bias/confounding so that the distributions of the observed pretreatment characteristics were similar across the different treatment groups at the follow-up at 3 months. Propensity score^17^ was defined as the probability of assignment to treatment condition based upon each participant’s set of observed risk factors potentially influencing the decision to re-engage at the check-in at 3 months. The propensity score for re-engagement was created using a multivariable logistic regression model which included participants’ demographic variables (age, gender, race, education level, marital status, military status, and baseline FTND scores), the first three-month treatment adherence (measured as number of counseling sessions attended and NRT use), and additional NRT or other medications use. The propensity score-adjusted logistic regression model was used to assess differences in past-7-day smoking abstinence rates at follow-up at 12 months between the treatment groups. The C- index^18^ was used as a measure of overall model predictive discrimination, defined in this study as the ability to differentiate participants who quit tobacco by the follow-up at 12 months from those who did not. Bonferroni multiple comparison adjustment was applied to control the type I error rate due to multiple comparisons. The overall significance level was specified at 0.05. All analyses were performed using SAS 9.4^19^.

## RESULTS

**Figure 1:**
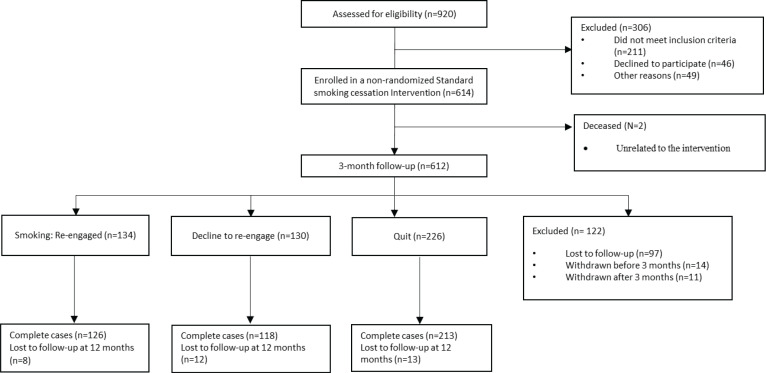
Consort diagram for the 2017–2020 Freedom Quitline (N=614)

Of 490 (79.8%) participants contacted at 3 months following a standard smoking cessation intervention, 226 (46.1%) reported abstinence and 264 (53.9%) were still smoking following an initial quit attempt (Figure 1). Of 264 who were still smoking, 134 (50.8%) were willing to re-engage for additional treatment.

Comparisons of demographic and tobacco use characteristics by smoking status at 3 months are presented in Table 1. There were differences among the three groups concerning race (p=0.033), military status (p=0.024), number of daily cigarettes smoked at baseline (p=0.010), FTND score (p=0.001) and NRT use, number of counseling sessions completed (p<0.0001), and additional medications used during the first 3 months of the study’s intervention period (p<0.0001).

**Table 1:** Characteristics of freedom quitline participants at baseline and at follow-up at 3 months, by smoking status, 2017–2020 (N=490)

*Characteristics*	*Smoking but re-engaged (n=134)*	*Smoking but declined to re-engage (n=130)*	*Abstinent (n=226)*	*p*
***Baseline***				
**Age** (years)	49.0 (34.0, 52.4, 63.0)	48.3 (32.6, 50.3, 62.8)	49.4 (34.4, 53.6, 62.0)	0.863
**Gender**				
Male	66 (49.3)	73 (56.1)	137 (60.6)	0.111
Female	68 (50.8)	57 (43.9)	89 (39.4)	
**Race**				
White	98 (73.1)	110 (84.6)	188 (83.2)	0.033
African American	24 (17.9)	14 (10.8)	26 (11.5)	
Other	12 (9.0)	6 (4.6)	12 (5.3)	
**Ethnicity**				
Non-Hispanic	120 (90.2)	121 (93.8)	198 (87.6)	0.176
Hispanic	13 (9.8)	8 (6.2)	28 (12.4)	
**Education level**				
High school diploma or GED	29 (21.6)	24 (18.5)	38 (16.8)	0.438
Some college/vocational school/associates degree	74 (55.2)	64 (49.2)	120 (53.1)	
Bachelor’s degree or post college	31 (23.1)	42 (32.3)	68 (30.1)	
**Military status**				
Dependent	60 (44.8)	45 (34.6)	67 (29.7)	**0.024**
Active	29 (21.6)	44 (33.9)	79 (35.9)	
Retired	45 (33.6)	41 (31.5)	80 (35.4)	
**Marital status**				
Single/widowed/divorced/separated	38 (28.4)	40 (30.8)	59 (26.1)	0.639
Married/living as married	96 (71.6)	90 (69.2)	167 (73.9)	
**Smoking attributes**				
Number of cigarettes per day	17.6 (10.0, 20.0, 20.0)	18.0 (10.0, 20.0, 20.0)	15.5 (10.0, 15.0, 20.0)	**0.010**
Years smoked	28.7 (13.0, 30.0, 42.0)	29.1 (15.0, 25.0, 44.0)	26.6 (13.0, 25.0, 40.0)	0.285
Fagerström score (FTND)	4.7 (3.0, 5.0, 6.0)	4.4 (3.0, 5.0, 6.0)	3.8 (2.0, 4.0, 5.0)	**0.001**
***Follow-up at 3 months***				
**Number of counseling sessions attended**	3.2 (3.0, 4.0, 4.0)	2.7 (2.0, 3.0, 4.0)	3.5 (4.0, 4.0, 4.0)	**<0.0001**
**NRT use**				
No	41 (30.6)	65 (50.0)	40 (17.7)	**<0.0001**
Yes	93 (69.4)	65 (50.0)	186 (82.3)	
**Additional medication use**				
No	15 (11.2)	53 (40.8)	19 (8.4)	**<0.0001**
Yes	119 (88.8)	77 (59.2)	207 (91.6)	

Categorical variables are displayed as frequency (%) and continuous variables are displayed as mean (1st quartile, median, 3rd quartile). Values of the p were derived from Fisher’s exact test for the categorical variables and Kruskal-Wallis test for the continuous variables.

The final propensity score-weighted logistic regression model demonstrated higher odds of past-7-day abstinence at the follow-up at 12 months for those who were abstinent at 3 months compared to those in the re-engaged group (OR=9.6; 95% CI: 5.2–17.8; Bonferroni adjusted p<0.0001) and compared to the group who declined to re-engage (OR=13.4; 95% CI: 6.8–26.8; Bonferroni adjusted p<0.0001) (Table 2). Notably, those who received only one course of treatment (i.e. including those who declined to re-engage and who were abstinent at follow-up at 3-months) were also more than 2.6 times likely to be abstinent at follow-up at 12 months than those who received two cycles of treatment (OR=2.6; 95% CI: 1.5–4.7, Bonferroni adjusted p=0.004). The re-engagement group did not have significantly higher odds of smoking abstinence at 12 months compared to those who still were smoking at 3 months, but who declined the offer to re-engage in a second cycle of treatment (OR=1.39; 95% CI: 0.68–2.85, p=0.367). The C-index of 0.77 from the final propensity score weighting logistic regression model indicated that this model had very good predictive discrimination power between participants who were and were not abstinent at follow-up at 12 months.

**Table 2:** Treatment effects of smoking status at 3 months on past-7-day abstinence at follow-up at 12 months, 2017–2020 freedom quitline participants (N=490)

*Comparison*	*OR (95% CI)*	*p*
Quit (74.9%)^a^ vs declined to re-engage (18.2%)	13.39 (6.81–26.32)	<0.0001
Quit (74.9%) vs re-engaged (23.7%)	9.63 (5.21–17.80)	<0.0001
Re-engaged (23.7%) vs declined to re-engage (18.2%)	1.39 (0.68–2.85)	0.367

The model was adjusted for the propensity score weighting to minimize potential selection bias at the follow-up at 3 months.

a Propensity scores adjusted past-7-day smoking abstinence rate at follow-up at 12 months.

For our analyses, we assumed that data not present at the follow-up at 12 months were missing at random (MAR). Only 33 of 490 participants at follow-up at 3 months were lost to follow-up at 12 months (about a 6.7% attrition rate for the second-phase treatment) (Figure 1). A propensity-score-weighted logistic regression analysis indicated that there were no differences in loss at follow-up at 12 months between the treatment groups (p=0.154). This additional sensitivity analysis provided evidence for the validity of the MAR assumption.

## DISCUSSION

In the current study, we evaluated the impact of proactive re-engagement on the performance of a tobacco Quitline, defined as past-7-day point prevalence at 12 months following the initial round of treatment. At follow-up at 3 months, 46.1% of participants had quit smoking and 50.8% of those who had not quit smoking were willing to re-engage in treatment. At follow-up at 12 months, 23.8% of re-engaged participants were abstinent, resulting in an overall study crude quit rate of 46.4%.

Such performance exceeds that associated with most multi-component, face-to-face smoking cessation interventions. The typical quit rate for Quitline interventions is 33%^20^, and we had anticipated that using proactive counseling to re-engage callers, whose initial quit attempt had failed, would improve cessation outcomes. Though effective in producing a meaningful number of additional quits, re-engagement was less successful than anticipated at improving cessation at follow-up at 12 months. We underestimated the rate at which Quitline callers would decline the opportunity to re-engage, and it is conceivable that a statistically significant treatment effect for re-engagement would have emerged had we achieved adequate statistical power.

Our observation that 24% of participants who agreed to re-engage were abstinent at 12 months supports the utility of proactive re-engagement for the 53.9% of participants who were still smoking three months following their initial quit attempt. Berman et al.^21^ have estimated that each smoker generates, on average, an additional $2056 in healthcare expenses annually. Thus, the 30 quits we enabled through re-engagement may have saved the Military Health System up to $62000 in the year following the study period. The value of this strategy is predicated upon an assumption that these callers would have continued smoking, had they not been given the opportunity to re-engage with treatment. Under such an assumption, overall Quitline performance would have attenuated by 6.6% in absence of re-engagement, and from this perspective the strategy appears to have resulted in a beneficial effect on the overall performance of the Quitline. The utility of re-engagement is further reinforced by our observation that callers who were still smoking at the check-in at 3 months but declined re-engagement, had an estimated quit rate 4.3% lower at 12 months than those who re-engaged.

Results from a comparison of callers who re-engaged with those who had achieved abstinence by the follow-up at 3 months were striking. We had anticipated that the re-engaged group’s receipt of additional treatment would render their cessation rate roughly commensurate with that observed for those who had achieved abstinence in the first three months of the study. Interestingly, those who were abstinent at the check-in at 3 months were nearly 10 times as likely as those who re-engaged to be abstinent at follow-up at 3 months. Propensity score adjustment allowed us to balance for confounding covariates, including baseline participant characteristics, severity of tobacco dependence, and the use of nicotine replacement therapy and completion of counseling during the study period. The fact that early abstinence outperformed re-engagement, even after the propensity score weighting adjustment, introduces the possibility that Quitlines wishing to incorporate re-engagement should consider investing in services (e.g. tailored text messages, stepped-care support) which maximize the probability of a quit within the first three months of treatment. These findings also suggest that future studies may benefit from evaluation of a broader array of re-engagement strategies tailored to specific groups of smokers.

Early success emerges as especially important in light of previous research demonstrating the diminishing likelihood of a successful quit following a failed attempt in the preceding year. Critically, this phenomenon strengthens with multiple such failed attempts at abstinence over the same period^22^. A Phase-Based Model^23^ for the structuring of tobacco cessation efforts seeks to optimize smokers’ prospects for a successful quit across a spectrum spanning from initial motivation, into preparation for behavior change, transversing the cessation attempt itself, and extending ultimately through the potential for relapse and recovery. Each of our participants was motivated to quit, having elected to call the Quitline and subsequently engaged with an intervention organized around the principles of motivational interviewing, and all callers participated in preparatory counseling broadly consistent with that outlined by Piper et al.^24^ . Emerging evidence for an additive effect of maintenance counseling in conjunction with extended use of NRT suggests that we may have achieved a better outcome for those who ultimately were not abstinent at three months during the ‘cessation’ phase – albeit at higher cost – had we interspersed the two months following the core intervention with bi-weekly phone contacts^25^. Latent class analysis has been used to successfully identify smokers for whom supplemental support may be indicated^26^, introducing the possibility that additional phone contacts could, in the future, be extended with increasing precision to smokers in need.

While nearly 70% of smokers wish to quit^27^, only a subset of especially motivated persons elects to engage with Quitline counseling on any given month^28^. It is thus critical that Quitline services be organized and executed in a manner which maximizes callers’ potential for successful tobacco cessation. Taken in context of the existing literature, our results raise the possibility that the right amount of Quitline treatment cannot be defined by a set number of calls irrespective of the individual, and should instead be understood in terms of each caller’s needs as anticipated by empirically-derived predictors of treatment response (e.g. perceived self-efficacy, and sleep status)^24^ and informed by their manifest response to treatment in the days and weeks following their target quit date. We extended an invitation to re-engage at three months, and this may have been as many as 90 days too late, depending upon the caller.

### Strengths and limitations

Strengths of the present study include treatment conditions featuring counseling and NRT consistent with established Clinical Practice Guidelines^29^, in addition to proactive engagement of callers following the initial Quitline contact, which thereby limited the influence of extraneous variables on callers’ ongoing engagement with the Quitline. Each of our counselors was trained to demonstrable proficiency in motivational interviewing using the MITI^16^ and received feedback on randomly selected audiotaped Quitline calls throughout the study period. Standardization of training and intervention delivery enabled isolation of variables, including re-engagement status, which were of central interest to the study.

This study was limited by a smaller than anticipated sample size – fewer participants than anticipated were willing to re-engage – which limited our ability to further investigate the degree to which treatment was less effective for more nicotine-dependent smokers, specifically. Also noteworthy is the exploratory nature of the analyses, residing within a larger trial specifically designed to evaluate the relative merits of three distinct re-engagement strategies. Consequently, what is, for the present purpose, referred to as ‘re-engagement’ comprises rate reduction, repetition of the earlier intervention, or the caller’s choice between the two, and these alternatives appear not to be commensurately effective. Caller tobacco use status also was ascertained by self-report (a valid method by which to assess smoking status when there is nothing to be gained from falsification of response), rather than empirically by means of salivary cotinine sampling. Finally, because we defined the one-year outcome as 12 months from completion of the first Quitline contact, we are limited in our ability to make strong inferences concerning the durability of quitting resulting from re-engagement, which occurred only 9 months in advance of the follow-up at 12 months.

## CONCLUSIONS

Our results suggest that, while re-engagement appears to be of benefit to those smokers for whom an initial round of treatment was unsuccessful, persons who succeed in achieving abstinence within that initial round of treatment are substantially more likely to be quit at 12 months relative to their re-engaged counterparts. Consequently, future research should seek to set on empirical footing a framework through which to tailor the amount and timing of supplemental tobacco cessation services inside the three months immediately following each caller’s target quit date, as success in this respect stands to increase considerably the corresponding quit rate at 12 months.

## Data Availability

The data supporting this research are available from the authors on reasonable request.
