# Supplementary material for: Tobacco quitline performance: Comparing the impacts of early cessation and proactive re-engagement on callers’ smoking status at follow-up at 12 months
Source: Tob Induc Dis. 2023 Feb 15;21:24. doi: 10.18332/tid/159125 (PMC9923459; doi:10.18332/tid/159125)
Supplement: Supplementary file 1 [file TID-21-24-s1.pdf]

FREEDOM Quit Line

Intervention Calls

You’ve been assigned a client

1. Contact your client within 24-48 hours to schedule your first call.
2. Be as flexible as you can with scheduling session calls, without impacting essential job tasks (i.e. Airmen briefings, etc.)…contact Freedom coordinator if you have problems in this area.
3. If no response from client, continue attempts to reach client on different days, different times, with different modalities (phone, text, email).

Sample Text:

I'm [Name], your Freedom Quitline counselor. I'd like to schedule a time to chat. What day and time is good for you?

Sample Email:

Hi [Name],

This is your counselor, [Name], from the Freedom Quitline. I’m trying to get a hold of you to schedule your first session. Let me know when would be a good time to chat. You can either call me at (XXX) XXX-XXXX or email me back when you get a chance. Really looking forward to speaking with you.
Thanks!
[Name]

Preparing for your session call

1. Block 15 minutes prior to call time to get prepared
2. Review screening call data and/or previous session notes
3. Review lesson material
4. Make the call

Baseline Sessions

Session 1: Engage/Rapport, Evoke Change Talk, Rate Reduction

Session 2: Pre-quit – Discuss quit date, manage triggers, 5A's, NRT use

Session 3: Post-Quit – Problem solving, Managing withdrawal symptoms, relapse prevention

Session 4: Relapse Plan - Problem solving, Managing withdrawal symptoms, relapse prevention

*Remind participants throughout these baseline sessions of the 3-month follow-up; schedule follow-up prior to last session

Rate Reduction Sessions

Session 1: Address former attempt, MI Discussion of Change, Rate Reduction techniques, nicotine gum use

Session 2: Progress check, Review Reduction techniques

Session 3: Progress check, Review Reduction techniques, Making a Quit Attempt

* Remind participants throughout these baseline sessions of the 3-month follow-up; schedule follow-up prior to last session

© 2023 Cassidy D.G.
